# Multi-scale temporal variation in bird-window collisions in the central United States

**DOI:** 10.1038/s41598-021-89875-0

**Published:** 2021-05-26

**Authors:** Corey S. Riding, Timothy J. O’Connell, Scott R. Loss

**Affiliations:** 1 Department of Natural Resource Ecology and Management, Oklahoma State University, 008C Ag Hall, Stillwater, OK 74078 USA; 2 Present Address: Department of Biology, Salt Lake Community College, 4600 South Redwood Rd, Salt Lake City, UT 84123 USA

**Keywords:** Conservation biology, Urban ecology

## Abstract

Expansion of urbanization and infrastructure associated with human activities has numerous impacts on wildlife including causing wildlife-structure collisions. Collisions with building windows represent a top bird mortality source, but a lack of research into timing of these collisions hampers efforts to predict them and mitigate effects on avian populations. In Stillwater, Oklahoma, USA, we investigated patterns of bird-window collisions at multiple temporal scales, from within-day to monthly and seasonal variation. We found that collisions peaked during overnight and early morning hours, a pattern that was consistent across seasons. Further, temporal variation in fatal collisions was explained by an interaction between season and avian residency status. This interaction illustrated the expected pattern that more migrant individuals than residents collided in fall, but we also documented unexpected patterns. For example, the highest monthly total of collisions occurred in spring migration during May. We also found similarly high numbers of resident and migrant collisions in spring, and a roughly similar amount of migrant mortality in spring and fall migration. These findings, which provide unprecedented quantitative information regarding temporal variation in bird-window collisions, have important implications for understanding mechanisms by which birds collide and improving timing of measures to reduce this major bird mortality source.

## Introduction

As earth’s human population grows, urbanization and the construction of infrastructure (e.g., buildings, roads, communication towers, and energy installations) are increasing. This expanding human footprint has many adverse effects on wildlife and the terrestrial, aquatic, and aerial ecosystems they inhabit^1–3^. Collisions of wildlife with vehicles and human-made structures are a major, increasing source of mortality associated with urbanization, particularly for volant animals like birds and bats^4,5^. Recognition of the increasing severity of wildlife collisions has led to growth in research evaluating the magnitude and effects of various collision sources, the factors driving collision rates, and the optimal approaches to reduce collisions in order to assist conservation efforts for affected species^6,7^.

For birds, collisions with buildings, particularly at windows and other glassy surfaces, represent the largest source of collision mortality in North America^8,9^. A large body of research now exists for this mortality source, including studies that: test approaches to deter collisions^10–13^; identify building-, vegetation-, and landscape-related correlates of collision rates^14–17^; and quantify and identify correlates of biases influencing collision estimates (e.g., imperfect searcher detection and scavenger removal of bird carcasses)^18–20^. Understanding the timing of bird-window collisions—for example whether birds collide more frequently in migratory or non-migratory periods or in the morning or afternoon—is important for understanding the likelihood of population impacts^21^, the ultimate mechanisms causing birds to collide (e.g., avian behavior and sensory traits, and external factors like weather), and the optimal timing of management interventions. However, relatively few studies have quantified the timing of bird-window collisions beyond anecdotal or descriptive accounts (but see ^18,22–25^), and none have done so at the multiple scales at which this temporal variation appears to occur.

Multi-scale, temporal variation in bird-window collisions is expected based on avian and vegetation phenology, bird behaviors that vary through time, and human behaviors and activity patterns that influence vegetation and bird behavior. Evidence suggests that both diel^26–29^ and seasonal^18,30–32^ collision patterns exist, as opposed to a random or uniform temporal distribution of collisions. Daily patterns are likely influenced by bird behaviors and activity schedules. Nearly all birds exhibit bimodal diel patterns in foraging and local movements, with the highest peak early in the day and a secondary peak in the evening^33,34^. For migratory species, diel patterns of long-distance movements also exist, with some species migrating primarily during the day, and others, including those most vulnerable to window collisions^9^, migrating primarily at night. This behavioral variation likely interacts with daily variation in human-related factors that influence collisions, such as reflectivity of glass panes in relation to the position of the sun. Anthropogenic lighting also exhibits strong diel variation and can attract and disorient nocturnally migrating birds^35^, which elevates collision risk during overnight and early morning periods^36,37^, despite migrating birds also being prone to window collisions in daylight hours during local (e.g., foraging) flights^15^. Likely due to a combination of the above factors, past studies suggest tentative diel patterns in collisions, such as most collisions appearing to occur between sunrise and noon during migration^26,38,39^ and between late morning and early afternoon in the breeding season^29^.

Seasonal collision patterns are likely influenced by avian life history strategies (e.g., year-round resident versus migratory)^29,30^; variation in weather, bird population sizes, and human provision of food at bird feeders near residences^24,32,40,41^; and phenology of vegetation that provides food, concealment, and/or nesting substrates near buildings. Overall, studies indicate that collision mortality tends to be higher during migratory periods, especially in fall migration (in the northern hemisphere)^9,18,25^. Geography may mediate such patterns by influencing the magnitude and timing of migration peaks at different latitudes^42^. For example, seasonal peaks of collision rates for migratory species should occur later in spring and earlier in fall with increasing latitude.

To enhance understanding of factors driving bird collision timing and provide information to improve collision deterrence efforts, we conducted an analysis of multi-scale temporal variation in bird-window collisions in a small urban area in the U.S. Great Plains. This region is relatively unstudied with respect to bird-window collisions. Further, small urban areas in largely rural landscapes, such as our study area, are understudied despite evidence that variation in and predictors of collisions in such settings differ from intensely urbanized regions^15^. Our specific objectives were to: (1) quantify diel (time-of-day) collision patterns across and within seasons by conducting morning, midday, and evening collision surveys, and (2) assess monthly and seasonal collision patterns, including in relation to avian residency status, by conducting collision surveys from April through October^43^. Based on evidence from past studies, and with respect to objective 1, we predicted collisions would occur most frequently during morning hours (i.e., most carcasses would be found during midday surveys) and that diel patterns would be consistent across seasons. With respect to objective 2, we predicted mortality would be highest in fall and that we would observe more collisions for migrant than resident individuals, both within migration seasons and combined across all seasons.

## Results

### Diel collision patterns

**Figure 1:**
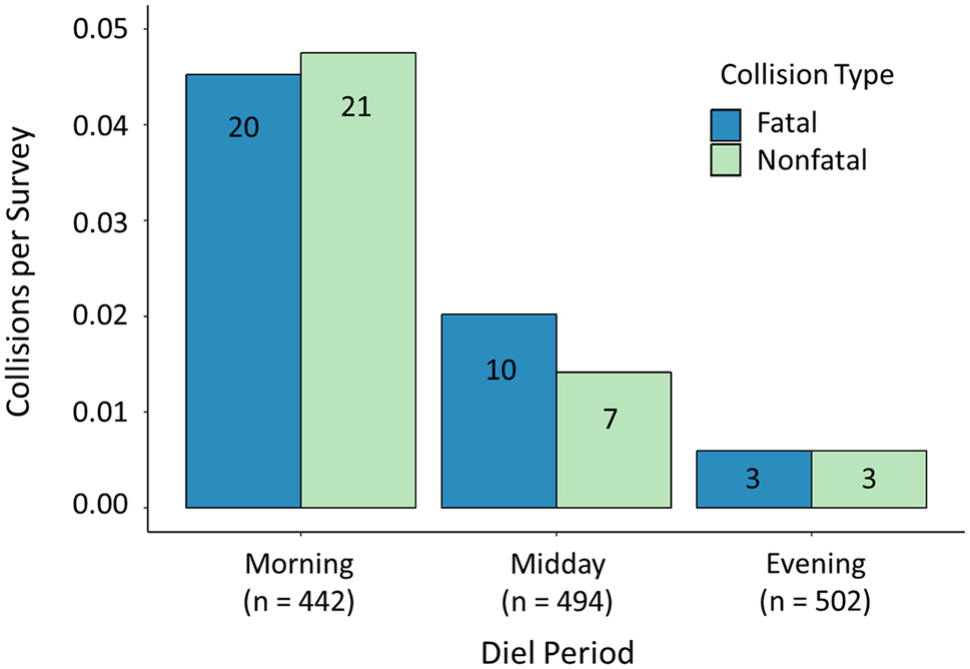
Number of bird-window collisions relative to time of day. Number of fatal and nonfatal bird collisions per survey during three different diel periods in Stillwater, Oklahoma, USA, 2015–2016. Total collision counts are shown in bars, and n is the number of surveys for each period.

For diel analyses, and across 5 buildings for which we monitored a portion of exterior glass surfaces, we conducted 1438 bird collision surveys (442 morning, 494 midday, and 502 evening surveys, with each survey representing a single building being searched once). In total, we tallied 33 fatal collisions from bird remains (i.e., carcasses or feather piles) and 31 non-fatal collisions (i.e., stunned birds or birds we observed to collide and then fly away) (Fig. 1, Supplementary Fig S1). Project volunteers conducted 44 (10%) morning surveys, but all midday and evening surveys were conducted by full-time technicians or the authors. We started very few surveys (n = 29; 2%) outside of our target time frames for each period, and all surveys started within ~ 60 min of either the beginning or end of the target frame. Further, intervals between successive surveys at the same building were always ≥ 120 min.

For the diel analysis of fatal collisions, ranking of zero-inflated Poisson (ZIP) regression models using Akaike’s Information Criterion corrected for small sample sizes (AICc) resulted in 3 models in the confidence set (i.e., ∆AICc ≤ 2; see Table 1 for confidence set models and Supplementary Table S1 for full model selection results). Among confidence models, the logit component included models containing a variable for survey start time (hereafter “SurveyTime”) or the intercept only (i.e., null model). Model averaging indicated that the number of structural zeros tended to increase with an increase in SurveyTime (i.e., more surveys with zero fatal collisions later in the day; β = 0.31, SE = 0.11); specifically, the odds of there being no fatal collisions found during a survey increased by a factor of 1.37 (the exponentiated coefficient of SurveyTime; i.e., *e*^0.31) for each hour later in the day. The count components in the confidence set included the model containing the variable for season (hereafter “Season”), the intercept-only model, and the SurveyTime + Season model. Notably, the interaction term SurveyTime*Season did not appear in the confidence set, which indicates there is little support for diel patterns of fatal collisions varying by season. Based on model-averaged coefficients, the number of fatal collisions found during surveys used for the diel analyses was lower in summer relative to fall (β = − 0.97, SE = 0.53) and decreased with an increase in SurveyTime (i.e., similar to the above logit model, fewer fatal collisions later in the day; β = − 0.21, SE = 0.06; Fig. 1). However, the number of fatal collisions did not differ substantially between spring and fall (β = − 0.07, SE = 0.45).

**Table 1:** Model selection results for analysis of diel variation in fatal bird-window collisions.

Logit model	Count model	K^a^	ΔAICc^b^	AIC weight^c^
SurveyTime	Season	7	0.0	0.49
SurveyTime	Null	5	1.0	0.30
Null	SurveyTime + season	9	1.6	0.22

Confidence set models (i.e., those with ΔAICc ≤ 2) resulting from full model selection comparison among candidate zero-inflated Poisson regression models that included factors potentially affecting numbers of fatal bird collisions found in morning, midday, and evening collision surveys in Stillwater, Oklahoma, USA in 2015–2016. Parameters in logit and count models included Season (spring, summer, fall) and SurveyTime (survey start time in decimal format). Weights are based solely on comparison of these models; full model selection results are shown in Supplementary Table S1.

^a^Number of model parameters.

^b^Difference in AICc value between model and top model.

^c^AICc Weight—relative support for model.

For the diel analysis of non-fatal collisions, AICc ranking of ZIP regression models resulted in 2 confidence set models, among which the only predictor variable to appear in either the logit or count components was SurveyTime (see Table 2 for confidence set models and Supplementary Table S2 for full model selection results). The top overall model had an intercept-only logit model with SurveyTime in the count model, while the other confidence set model had SurveyTime in the logit model and an intercept-only count model. This suggests that only SurveyTime was important to both the number of structural zeros and the number of non-fatal collisions, but it also suggests the influence of SurveyTime on non-fatal collisions may be weak. As described fully in the methods, we were unable to test for an interaction between SurveyTime and Season for non-fatal collisions due to model convergence issues. Model averaging of the confidence set indicates that structural zeros increased with SurveyTime (i.e., more surveys with zero non-fatal collisions later in the day; β = 0.31, SE = 0.09) and that the number of non-fatal collisions decreased with an increase in SurveyTime (i.e., fewer non-fatal collisions later in the day; β = − 0.25, SE = 0.06; Fig. 1).

**Table 2:** Model selection results for analysis of diel variation in non-fatal bird-window collisions.

Logit model	Count model	K^a^	ΔAICc^b^	AIC weight^c^
Null	SurveyTime	5	0.0	0.69
SurveyTime	Null	5	1.6	0.31

Confidence set models (i.e., those with ΔAICc ≤ 2) resulting from full model selection comparison among candidate zero-inflated Poisson regression models that included factors potentially affecting numbers of non-fatal bird collisions (including stunned birds or birds we observed to collide and then fly away) found in morning, midday, and evening collision surveys in Stillwater, Oklahoma, USA in 2015–2016. Parameters in logit and count models included Season (spring, summer, fall) and SurveyTime (survey start time in decimal format). Weights are based solely on comparison of these models; full model selection results are shown in Supplementary Table S2.

^a^Number of model parameters.

^b^Difference in AICc value between model and top model.

^c^AICc Weight—relative support for model.

### Monthly and seasonal collision patterns

**Figure 2:**
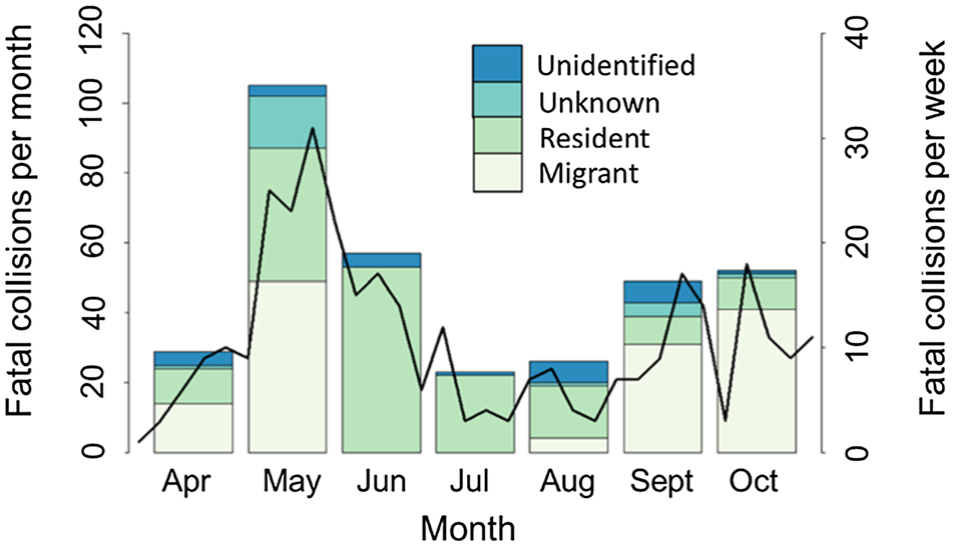
Monthly and weekly variation in bird-window collisions. Monthly (left vertical axis and bars) and weekly (right vertical axis and black line) numbers of fatal collisions (including feather piles) by residency status of individual birds from collision monitoring in Stillwater, Oklahoma, USA in 2015–2016. See “Methods” for details regarding designation of residency status groups.

For monthly and seasonal analyses, we conducted 6631 surveys of the perimeters of 16 buildings between April and October. We also conducted 350 surveys between November and March but did not formally analyze these data because sampling methodology differed (see “Methods”), which likely contributed to us observing fewer carcasses (n = 19) and feather piles (n = 3) compared to other months (Table 3). For surveys from April to October, we found 341 fatal collisions (275 carcasses and 66 feather piles); in Fig. 2, we illustrate monthly and weekly patterns of fatal collisions, including for each residency status category (i.e., whether an individual bird was in migration or a resident/sedentary period of its annual cycle; hereafter “ResStatus”). Overall, the highest monthly total of collisions occurred in spring migration during May due to a relatively high number of collisions for both migrant and resident individuals (Fig. 2). June had the second highest raw count of total of fatal collisions, which consisted almost entirely of resident birds. However, when we adjusted fatality counts for rates of scavenger removal and searcher detection, June ranked slightly behind both September and October, fall months in which the majority of collisions were of migrants (Table 3). Over the entire study period, we found more carcasses of resident birds (148) than migrants (139). Because we sometimes conducted multiple surveys per day, the apparently low frequency of observed collisions (fatal or non-fatal collisions found on 5.1% of Apr-Oct surveys) should not necessarily be interpreted as a low collision rate in the context of other studies that quantified bird collision rates on a per building and/or annual basis. Such data on building-level collision rates in our study area are presented elsewhere^20,43^.

**Table 3:** Results of bird-window collision surveys. Raw counts and bias-adjusted estimates of fatal collisions by month based on collision surveys in Stillwater, Oklahoma, USA in 2015–2016.

Month	Number of surveys	Number of carcasses	Number of feather piles	Total count	Seasonal bias	Adjusted carcasses	Adjusted total count	Adjusted total per survey
Jan	56	0	1	1	0.680	0.0	1.5	0.027
Feb	56	0	0	0	0.680	0.0	0.0	0
Mar	79	3	0	3	0.680	*	*	*
Apr	982	24	5	29	0.883	27.2	32.8	0.033
May	1004	92	13	105	0.883	104.1	118.9	0.118
June	879	41	16	57	0.905	45.3	63.0	0.072
July	985	17	6	23	0.905	18.8	25.4	0.026
Aug	925	13	13	26	0.905	14.4	28.7	0.031
Sep	886	39	10	49	0.758	51.4	64.6	0.073
Oct	970	49	3	52	0.758	64.6	68.6	0.071
Nov	94	15	2	17	0.680	*	*	*
Dec	65	1	0	1	0.680	1.5	1.5	0.023

Bias adjustments incorporate seasonal estimates of searcher efficiency and scavenger removal rates of bird carcasses, and adjusted total counts are based on applying bias adjustments to raw counts excluding feather piles.

*Surveys in March and November occurred more frequently than calculated in bias adjustments, so adjusted values would be overestimated for those months.

**Figure 3:**
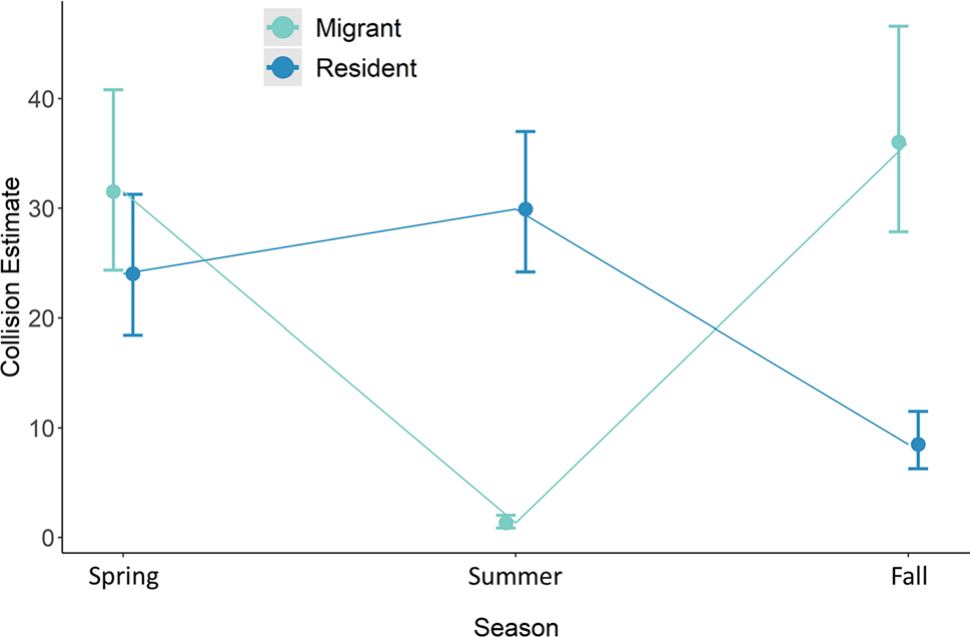
Interacting effect of season and residency status on variation in bird-window collisions. Interaction plot illustrating model-predicted numbers (± standard errors) of collisions for different combinations of residency status (i.e., whether individual birds that collided were migrants or residents) and season (spring, summer, fall). This interaction was the only predictor variable in the only confidence set model (i.e., ΔAICc ≤ 2) in an analysis of factors associated with raw counts of total fatal bird collisions (including feather piles) in Stillwater, Oklahoma, USA in 2015–2016 (model selection results in Table 4).

Results of analyses of seasonal and monthly collision variation were nearly identical for three different dependent variables (raw counts of fatal collisions including feather piles, raw counts excluding feather piles, and raw counts excluding feather piles and adjusted to account for searcher detection and scavenger removal of carcasses), with models ranked in the same relative order and estimated coefficients having the same sign and very minor estimated differences in effect sizes (see Supplementary Tables S3, S4). Therefore, we present only results for the analysis of raw fatal collisions including feather piles. For this analysis, there was only one confidence set model (Table 4), which contained the interaction between Season and ResStatus. The interaction plot for this model (Fig. 3) indicates that resident and migratory individuals collided in approximately equal high numbers during spring, residents collided far more often than migrants during summer, and migrants collided more often than residents in fall. Another way to express this interaction is that migrants collided in roughly equal high numbers during spring and fall migration, whereas residents collided in roughly equal high numbers in spring and summer but in much lower numbers in fall.

**Table 4:** Model selection results for analysis of seasonal/monthly variation in bird-window collisions.

Parameters	K^a^	ΔAICc^b^	AIC weight^c^
Season * ResStatus	7	0.0	> 0.99
Null	2	105.8	< 0.01
Season	4	108.2	< 0.01
Month	3	108.5	< 0.01
Season + ResStatus	5	108.9	< 0.01
ResStatus	3	108.9	< 0.01
Month * ResStatus	5	110.2	< 0.01
Month + ResStatus	4	112.5	< 0.01

Results of model selection comparison among candidate negative binomial models that included factors potentially affecting raw counts of total fatal collisions (including feather piles) found in morning, midday, and evening collision surveys in Stillwater, Oklahoma, USA in 2015–2016. Parameters included Month (4 = April, 5 = May, etc.), Season (spring, summer, fall) and ResStatus (residency status of individual birds: resident and migrant birds were included in analysis; individuals of unknown residency status and of = unidentified species were excluded from analysis).

^a^Number of model parameters.

^b^Difference in AICc value between model and top model.

^c^AICc Weight—relative support for model.

In addition to seasonal and monthly patterns, we also observed week-to-week variation in collisions that, although generally coinciding with monthly trends (Fig. 2), indicated that collision variation occurred at a temporal scale intermediate to diel and monthly variation. For example, summarizing fatal collisions by week illustrated a small peak in collisions in early July and a relative lull in collisions in early October.

## Discussion

In this multi-scale assessment of temporal variation in bird-window collisions, our predictions related to the diel timing of collisions were only partly supported. We predicted more casualties would occur during morning than other times of day, which should have resulted in our detection of the greatest number of fatal collisions on midday surveys and more non-fatal collisions during morning surveys than midday and evening surveys. However, greatest numbers of both fatal and non-fatal collisions were observed on morning surveys, indicating that more collisions occurred during overnight and early morning periods than mid-to-late morning and afternoon. As predicted, this diel pattern was consistent across seasons. Our predictions about monthly and seasonal patterns were also only partly supported. Unexpectedly, total collision mortality was highest in the spring migration month of May, and avian residency status interacted with season such that roughly equivalent high numbers of resident and migrant collisions occurred in spring, more resident than migrant collisions occurred in summer (and overall from Apr to Oct), and more migrant than resident collisions occurred in fall. Further, unlike many past studies, collision mortality of migrants was roughly equivalent in spring and fall migration.

### Diel collision patterns

We observed more fatal and non-fatal collisions in the morning than in midday and evening surveys combined, even though we included fewer morning surveys in diel analyses. These differences are likely conservative in that an even greater proportion of fatal collisions than we observed likely occurred overnight and in the early morning, but went undetected because of bias associated with observer detection and scavenger removal of carcasses. Concurrent work in our study area^20^ found that relatively inexperienced volunteers detected a slightly lower proportion of carcasses (0.69) than experienced surveyors (0.76). Because roughly 10% of morning surveys were conducted by less-experienced volunteers and all midday or evening surveys were conducted by full-time technicians or the authors, we likely missed more carcasses on morning surveys. Additionally, most scavenging events (68%) were at night^20^, so bird carcasses from overnight collisions were the least likely to persist until the subsequent survey. Thus, underestimation of fatal collisions in the preceding interval was almost certainly greater for morning surveys than for midday and evening surveys.

A prevailing hypothesis for why daytime bird-window collisions occur is that birds making local (e.g., foraging) movements fail to perceive a barrier when flying toward objects either on the other side of glass or reflected on a glassy surface^8,15^. Under this hypothesis, daytime collisions for both residents and migrating birds at stopover locations would be expected to occur most frequently when birds are most active, which is typically near dawn regardless of season. Our finding of the greatest number of collision fatalities on morning surveys circumstantially supports the above hypothesis and expectation, as do past descriptive studies of diel variation in bird-window collisions^26,27,38,39^. However, our study design did not allow differentiation between nocturnal and early morning collisions, and a nontrivial proportion of carcasses detected on morning surveys likely represented collisions from the preceding overnight period. Nighttime collisions may occur at any structural component not easily detectable at night (i.e., they are not limited to glass surfaces), and can be exacerbated by artificial light emission that attracts and disorients migrating birds^36,37,44,45^. Nonetheless, the observation of more non-fatal collisions (including directly witnessed collisions) during morning than midday or evening surveys does strongly suggest that morning carcass counts included many collisions that occurred near or shortly after dawn.

A potential limitation of our study regarding time-of-day analyses is the longer interval between evening and morning surveys than between other survey periods. If collisions occurred uniformly or randomly in time, we would expect to find more bird carcasses during morning surveys due to the longer preceding time interval. However, as described above, the early morning peak observed for non-fatal collisions (Fig. 1), which are less persistent than carcasses and therefore do not accumulate over time, suggests that collisions do not have a uniform or random temporal distribution and that a real peak in collision frequency occurs sometime shortly prior to when we conducted morning surveys. Another possible bias is that we conducted surveys during fixed time periods rather than adjusting them to seasonally varying sunrise and sunset times. This limitation would have most strongly affected our summer results, potentially inflating summer morning counts as a result of collisions for both early morning and late evening (the two periods when birds are most active) being grouped into the same survey period. However, this limitation was unlikely to greatly influence our conclusions about diel collision patterns because: (1) these patterns were fairly consistent across seasons, suggesting a relatively small influence of varying sunrise and sunset times, and (2) sunrise and sunset times vary by only a few hours over the course of the entire year whereas the broad time periods for which we compared collisions (overnight, morning, afternoon) consist of longer lengths of time. Further research is needed to identify the exact timing of collisions, including during overnight periods, and this could be accomplished with collision surveys conducted more frequently during the day and night or remote detection methods, such as video cameras, motion-triggered still cameras, microphones that record sounds of impact, and glass-mounted pressure sensors that detect vibrations from collision impact.

We predicted diel collision patterns would not vary seasonally because the pattern of birds being most active near sunrise and sunset is relatively consistent across seasons. Hager and Cosentino^46^ provide excellent guidelines for conducting bird-window collision surveys, but their recommendation to conduct surveys in mid-to-late afternoon is based on summer monitoring that found mortality to peak between late morning and early afternoon in Illinois, USA^29^. We suspect differences in diel patterns between that study and ours relate to geographic variation and/or seasonal sampling coverage, as our larger sample of surveys included spring and fall migration in addition to summer. Although many collision-prone species migrate nocturnally, the diel collision peak for migrants could still occur in early morning because nocturnally-migrating birds often set-down into stopover habitats during early morning^47,48^ and may be most susceptible to collisions at this time. There could be subtle seasonal variation in diel collision patterns that we failed to detect; however, the majority of collisions across seasons appear to occur near or before dawn (see also^26,27,38,39^). In combination with the previous study showing that scavenging peaks overnight^20^, we suggest that conducting daily collision surveys in the morning could result in the least biased mortality estimates, especially in urbanized areas where humans (e.g., cleaning crews) may remove carcasses in the early morning. As noted by Hager and Cosentino^46^, further research is needed to identify how the optimal survey time is influenced by factors such as geography, the bird community, animal scavengers, and removal of bird carcasses by humans.

### Monthly and seasonal collision patterns

We expected more collisions in fall than other seasons because bird populations in North America are larger after summer breeding and include higher proportions of young birds that have less experience with flight, migration, and human structures. Also, numerous studies have found the greatest window collision mortality in fall, a pattern driven largely by migrant birds ^12,18,23,27,31,40,49^. Contrary to expectation, both raw counts and bias-adjusted estimates of collision fatalities were highest in the spring migration month of May and higher overall in spring than fall. This pattern resulted from a high number of both migrant and resident bird collisions. In fact, we found that roughly equal numbers of resident and migrant collisions occurred in spring. When considering migrating individuals only, we found roughly similar numbers of collisions in spring and fall migration, a finding that also is unexpected in light of past studies. Notably, two other studies that found that a large proportion of total collisions consisted of resident birds^30,50^ also documented a seasonal pattern of total collisions that was less skewed toward fall. An explanation for the relatively large amount of total spring mortality, and for our finding that migrant mortality was roughly similar in spring and fall, is that some long-distance migrants follow elliptical migration paths where migration routes in fall are farther east than in spring^51,52^, such that in central North America, numbers of some species are greatest during spring migration. This explanation is supported by our observation of some elliptical migrants colliding during spring but not fall (e.g., Swainson’s Thrush [*Catharus ustulatus*]). Our study adds further nuance to the understanding of seasonal variation in bird collisions and exemplifies the need to study bird-window collisions in a wider array of geographic contexts to allow region-relevant management recommendations.

Our predictions regarding avian residency status were only partly supported; more migrants than non-migrants were indeed killed during fall migration. In spring, however, roughly equal numbers of each group collided, and far more residents than migrants collided in summer (and overall from Apr–Oct). This result does not account for varying abundance (overall and seasonally) of each species group, so it does not necessarily imply that resident species are more vulnerable to, or at greater risk of, window collisions relative to their abundance or period of residency in our study area. However, the finding of a high proportion of non-migrant casualties was still unexpected given that previous similar studies have almost universally reported higher mortality among migrants^15,25,26,39,49,53,54^, although most sampled during migratory periods only. Even with our individual-based residency designations, we may have slightly underestimated migrant mortality because all individuals of some migratory species were classified as unknown due to overlapping resident and migratory periods. However, even if all unknown individuals were migrating, there were too few birds in this category (22 of 341 [7%] total carcasses) to change our conclusions regarding the migrant-resident comparisons. Anecdotally, many spring and summer collision fatalities were recently fledged juveniles (n = 26 [25%] in May; n = 17 [30%] in June), clearly indicating that some collision victims were indeed not migrating, and therefore, that the high number of resident collisions is not an artifact of our classification system. Moreover, we did not observe collisions of any migrant individuals during June, even though a few species migrate through our study area in small numbers during this period (e.g., shorebirds [order Charadriiformes] and some tyrant flycatchers [family Tyrannidae])^55^. It is possible, however, that some resident individuals were undergoing post-breeding dispersal at the time of collision, as evidenced by a small late-June peak of Tufted Titmouse (*Baeolophus bicolor*) and Carolina Chickadee (*Poecile carolinensis*) collision victims with brood patches (TJO unpublished data).

Although our sampling captured the peak months of spring and fall migration in our study region, we undoubtedly missed some early-spring migrants before April and late-fall migrants after October. However, greater than 10 years of near-daily collision surveys at one of the most collision-prone buildings in our study (TJO unpublished data) suggests this number of missed collisions was relatively small. Specifically, total collisions at this building (including residents and migrants) dropped from an average of > 8 birds in October to < 3 birds in November, with < 0.5 birds per month between December and March. Our surveys between November and March did document a non-trivial number of collisions (22 fatal and non-fatal collisions over 2 years) despite these surveys being conducted less frequently and at a smaller subset of buildings compared to monitoring from April to October. Based on these observations, future research is warranted to quantify relative numbers of collisions for migrant and resident individuals throughout all migration periods, and of total collisions throughout the year.

Factors related to avian reproductive phenology likely contributed to the monthly and seasonal collision patterns we observed. The relatively large number of collisions for resident birds from May to August likely reflects the main breeding period for these birds. During breeding, adult birds make foraging flights to supply food for nestlings and fledglings, and these frequent flights may increase collision susceptibility relative to non-breeding periods. Notably, adult fatalities experienced immediately before or during the breeding season may also have a disproportionate effect on population abundance because there is little time for population-level processes (e.g., density dependent increases in adult survival) to compensate for mortality prior to the breeding season^21^, and because dependent offspring may also die if adults are killed during the nestling or fledgling periods. In addition to collisions experienced by breeding adults, and as noted above, we observed many collisions of recently fledged juveniles and of adult birds likely undergoing post-breeding dispersal or dispersal between breeding attempts; these observations further support that breeding-related activities influence window collision risk.

Weather also was likely to contribute to the temporal variation we observed, especially for week-to-week variation in collisions. Weather conditions have been shown to influence building collisions for one species, the American Woodcock (*Scolopax minor*)^56^. Although formal analyses of weather effects on total bird collisions are lacking, weather appears to have contributed to several major bird collision events, including at buildings^45,57,58^ and other types of structures^59^. Weather conditions such as precipitation, cloud cover, and the presence and/or strength of headwinds or tailwinds—as well as extreme weather events like intense storms—are known to influence the timing and magnitude of bird migration^35^, and are thus likely to influence collision risk. Some of these factors (e.g., low cloud ceilings) may have especially strong effects on nocturnal migrants by exacerbating effects of nocturnal lighting and driving birds into areas of greater collision risk^32,56^. When considering the week-to-week variation we observed within seasons, the fine-scale peaks and lulls in collisions may respectively reflect weather conditions that favor or disfavor bird migration (e.g., strong tailwinds or headwinds, respectively) and/or elevate or reduce collision risk for migrating birds (e.g., low cloud ceilings or clear skies, respectively). A complementary explanation for weekly variation is the varying migration phenologies of different bird groups, e.g., fall collision peaks in late Sep and mid-Oct might reflect migration peaks for wood warblers (family Parulidae) and sparrows (family Passerellidae), respectively, in our study region. Finally, some of this short-term collision variation might have arisen simply due to stochastic factors unrelated to weather or the migration phenology of different species groups. Further research is needed to investigate correlations between weather and collisions at various temporal scales, and analyses such as ours that document multi-scale temporal variation in collisions contribute to clarifying these relationships.

## Conclusion

We documented multi-scale temporal variation in bird-window collisions, including diel, monthly, and seasonal patterns. This information is crucial for improving understanding of the mechanisms by which birds collide and for efficiently targeting collision reduction measures in time. For example, we found strong evidence that both fatal and non-fatal collisions peak overnight and/or during early morning hours. This pattern has previously been described for bird collisions with skyscrapers in major cities, and in a few instances with smaller buildings in small cities^38,39^. However, our study provides unprecedented quantitative support for this pattern, thus indicating that any temporary efforts to deter collisions (e.g., closing blinds, raising movable screens, emitting sonic deterrents)^12,60^ will likely be most effective during overnight and early morning periods. This pattern also suggests collision reductions may be achievable by enacting lights-out programs at smaller buildings and in smaller cities than for which this method has traditionally been prescribed.

We also found collisions to vary seasonally, monthly, and weekly. Although we documented the expected pattern that more migrant birds than resident birds collided in fall, we documented other unexpected patterns, including the highest monthly total of collisions occurring in the spring migration month of May, roughly equivalent high numbers of resident and migrant bird collisions in spring, and a similar amount of migrant mortality in spring and fall migration. At seasonal, monthly, and weekly temporal scales, weather, reproductive phenology, and other seasonal changes likely drive collision variation, thus predictions of collision risk based on weather and date may allow better focusing of collision deterrence efforts^56^. Our results can also inform sampling protocols for future studies of bird-window collision. Specifically, our findings of the greatest number of carcasses on early morning surveys, as well as the relatively high number of spring collisions, indicate that studies seeking to capture a larger and/or more accurate representation of birds killed should consider sampling during early morning and in both spring and fall migration.

Finally, future research could include multi-year monitoring to detect any longer-term collision variation. Quantifying annual or even longer-term variation in bird-building collision rates could help validate the patterns we documented in this study, detect changes in bird population abundances (e.g., lower collision rates could indirectly indicate population declines in response to collisions and other anthropogenic threats), and clarify whether birds have the potential to adapt to this relatively novel anthropogenic threat (e.g., if collision frequency decreases for species with stable or increasing abundance). Comprehensively understanding short- and long-term collision variation will be important not only for more effectively managing bird-building collisions but also for understanding effects of other anthropogenic disturbances to birds, including urbanization, land use and land cover changes, and climate change.

## Methods

### Study area and study design

We searched for evidence of bird-window collision events in Stillwater, Oklahoma, USA, an urban area with a human population of 45,688 in the 2010 census, covering an area of 73.3 km^2^, and located in a predominantly rural landscape. The study area lies in the Cross Timbers ecoregion, a transitional zone in the eastern Great Plains where oak and juniper woodland are interspersed with perennial grasslands. We selected survey buildings based on building size, amount of surrounding vegetation, and accessibility (see Hager et al.^15^, a continental study that included a subsample of our study buildings). Buildings varied in footprint area (200–8000 m^2^) and height (6–27 m), but none were the high-rise skyscrapers typical of large cites. We monitored 16 buildings, including residences (n = 2), buildings on the Oklahoma State University main campus (n = 11), and commercial off-campus structures (n = 3).

To serve as a baseline for assessing temporal variation in collisions, we conducted morning surveys around all buildings ≥ 6 days/week from April to October in 2015 and 2016, as described in^43^. We started these near-daily surveys between 0700–0900 h (all times Central), unless inclement weather or other extenuating circumstances (e.g., safety or volunteer availability) made this infeasible. Between November and March, we did not conduct full-scale monitoring due to staffing limitations and because bird-window collision mortality during this period is generally minimal compared to other months^22,23,25,31^, including in our study area, as evidenced by ~ 10 years of near-daily monitoring at one building included in this study (TJO *unpublished data*; see “Discussion” for details). We did monitor a subset of 4 buildings 1 day/week between November and March of 2015–2016 and 5 buildings 2 days/week during these same months in 2016–2017, but we excluded Nov–Mar data from formal statistical analyses because building selection and sampling intensity differed substantially from other months.

To assess diel patterns, we also conducted midday (1200–1400 h) and evening (1700–1900 h) surveys at a subset of the buildings monitored in the morning. To ensure an adequate sample size of collisions for diel analyses, these midday and evening surveys were conducted at non-randomly selected buildings or portions of buildings that we considered likely to experience the greatest number of collisions based on preliminary observations and putative correlates of mortality risk (e.g., large surface area of glass). We conducted these midday and evening surveys in 2-week (2015) or 1-week (2016) bouts within seasons, totaling 5 bouts in spring (Apr–May), 3 in summer (Jun–Aug), and 3 in fall (Sep–Oct). During these bouts, both midday and evening surveys were conducted for each day that morning surveys were conducted (i.e., ≥ 6 days/week).

### Data collection

Collision surveyors fell into two groups: experienced personnel who regularly and frequently conducted surveys (including the authors, full-time research technicians, and more experienced volunteer observers), and less-experienced volunteer community scientists who conducted surveys irregularly and infrequently (we describe in the Discussion how this variation in survey experience could have influenced results). Prior to participation, we required all surveyors to receive training on protocols for conducting surveys, collecting dead birds, and recording and entering data. During surveys, we walked slowly along the exterior perimeter of focal buildings, intensively searching a 2 m swath along walls with glass surfaces, such as windows. We entered three buildings to survey ledges below windows that could not be observed from outside. All surveys consisted of a single pass around each building or along each building segment, but we alternated the direction each building or segment was monitored daily (clockwise on even days, counter-clockwise on odd days).

The purpose of these surveys was to detect and accurately count fatal and non-fatal bird collisions. We did not include smudges (e.g., feathers or other bird-related markings on glass surfaces), as these had ambiguous outcomes and could have led to double-counting some collisions (e.g., one or more smudges in one location corresponding to a living or dead bird that moved to another location before being encountered). For our purposes, fatal collisions included both whole bird carcasses, as well as remains indicative of a bird carcass, which in most cases consisted solely of feathers (i.e., a feather pile) that had been plucked from the carcass by a scavenger. To avoid counting adventitiously lost feathers, we defined a feather pile to consist of ≥ 5 feathers within a circular area approximately 15 cm in diameter. As some feather piles could have originated from sources other than window collisions (e.g., predation of live birds), counts of feather piles were excluded from some analyses (as described below). We also recorded non-fatal collisions, including those directly witnessed by the surveyor where the bird did not immediately die and/or flew away after experiencing no apparent harm, and stunned birds lying on the ground or in vegetation that had likely suffered a recent collision. Surveyors recorded the location and a description of each collision event (including species, if known) and took photographs of carcasses and stunned birds. We collected carcasses and remains using a plastic, sealable bag, and subsequently stored them in a freezer with a unique alphanumeric identification code for each individual bird. When we could not collect a carcass (e.g., because it was on an inaccessible ledge above ground level), and at one building where carcasses were regularly left in place as part of a concurrent study^20^, we tracked the condition and location of the carcass to avoid double counting it on future surveys. We collected bird carcasses and remains under federal (U.S. Fish and Wildlife Service permit #MB05120C-0) and state (multiple permits over course of the study) scientific collecting permits with protocols approved by the OSU Institutional Animal Care and Use Committee (Animal Care and Use Protocol #AG-14-8). Other than attempting to photograph birds that collided for identification and documentation, we did not interact with live birds during surveys and were not required to obtain a separate Animal Care and Use Protocol. We followed published guidelines^61^ for best practices to minimize potential negative impacts to live birds during our surveys.

We determined residency status of each individual bird observed in collision surveys based on the date collision events were observed; the age of the bird, when determinable (e.g., hatch year birds are unlikely to be migrating in May and June); seasonal occurrence data from eBird^62^; and a guide to arrival, migration, and departure dates for species in our study region^55^. We categorized each collision victim as: (1) resident, for individuals from non-migratory species and seasonally or partially resident species found outside of a migratory period; (2) migrant, for any individual determined to be on migration, including summer and winter residents during their migratory periods and passage migrants that occur in our study area only while migrating; (3) unknown, for individuals from species (including partial migrants) with significant overlap in timing for resident and migratory periods (e.g., American Robin [*Turdus migratorius*] during Apr and Oct); or (4) unidentified, for any bird remains that could not be identified to species, most of which were feather piles.

### Statistical analyses

For diel analyses, we did not include surveys in analyses if the building where we found the bird was not surveyed during the preceding period (e.g., we excluded data from a morning survey if the same building was not surveyed the evening before). This was done to ensure we counted birds that collided only in the interval immediately preceding the survey. Excluding such surveys, and also a small number of surveys that we missed due to logistical constraints (e.g., safety-related issues associated with severe weather), resulted in slightly different numbers of surveys for each period (442 morning, 494 midday, and 502 evening surveys; each survey represents a single building being searched for carcasses once). For monthly/seasonal analyses, we included carcasses found during morning surveys, and we also included carcasses from midday and evening surveys because we assumed they would have been detected on subsequent morning surveys due to relatively low daily scavenging (0.09) and high surveyor detection (0.73) probabilities in our study area^20^.

We conducted all analyses in R 3.5.0^63^ with RStudio 1.1.447^64^. Where noted below, we tested for overdispersion of data using the function ‘dispersiontest’ in R package ‘AER’; these tests were conducted for global models without interaction terms that were fit using function ‘glm’. To compare and rank models for diel and monthly analyses (see below), we used Akaike’s Information Criterion corrected for small sample sizes (ΔAICc)^65^. We interpreted variables from models with strong support (ΔAICc ≤ 2) via conditional model averaging (function ‘model.avg’ in R package ‘MuMIn’), but we did not consider models that were more complex versions of higher ranking nested models (i.e., models that contained uninformative variables^66,67^).

To assess diel patterns, we treated individual surveys as replicates and separately analyzed two dependent variables (number of fatal collisions and number of non-fatal collisions). We used zero-inflated Poisson (ZIP) regression (function ‘glmmTMB’ in R package ‘glmmTMB’ with family = poisson) because the models were not overdispersed but > 97% of these surveys resulted in a count of 0 collisions. ZIP regression models are commonly used in cases of excess zero counts and have two parts: a logit model for predicting excess (structural) zeros and a Poisson model for predicting the count, which may or may not be zero^68^. We included an offset for number of surveys (specific to each season and building combination) to account for varying sampling effort. Also, we included year and building as random effects^69^ because the substantial variation across levels of these categorical variables was not of primary interest for our objectives. Notably, although inter-annual variation in collisions is likely to occur, our study was only 2 years long, which limits meaningful ecological inferences about patterns and correlates of such variation. Additionally, although the monthly analysis used fatality estimates adjusted for searcher detection and scavenger removal biases (see next paragraph), we were unable to do this for diel analysis—where individual surveys were treated as replicates—due to computational challenges of applying bias corrections to the results of individual surveys. Potential predictors for both the logit and count components of the ZIP model included Season (categorical: spring, summer, fall), SurveyTime (survey start time in decimal format where, for example, 7.5 = 0730 h), and the interaction Season*SurveyTime. However, when modeling the number of non-fatal collisions, we considered univariate logit models only because the algorithm often failed to converge with more than one variable or with interactions in that model component. We modeled the continuous SurveyTime predictor rather than a categorical Period predictor (coded numerically: 1 = morning, 2 = midday, 3 = evening) because SurveyTime and Period were highly correlated (Spearman r = 0.94), and we experienced fewer model convergence issues when using SurveyTime.

To assess monthly and seasonal patterns of fatal collisions, we summed counts for each month and residency class combination (i.e., month-residency class combinations treated as replicates), and we only included birds that were known migrants or residents (i.e., we excluded “unknown” birds that could have been either migrants or residents and “unidentified” birds that could not be identified to species). We separately analyzed three different dependent variables: (1) fatal collisions including both carcasses and feather piles, (2) carcass counts excluding feather piles, and (3) carcass counts excluding feather piles and adjusted for two major survey-related biases that cause raw counts to underestimate mortality: imperfect observer detection of carcasses and removal of carcasses between surveys by humans and animal scavengers. As described in detail elsewhere^20,43^, this adjustment was based on data from concurrent experimental removal and detection trials conducted at the same buildings during all seasons of monitoring. For removal trials, we placed previously collected bird carcasses and monitored them using trail cameras and daily surveyor checks until carcasses were removed or decomposed such that no visible remains persisted. For detection trials, we placed separate bird carcasses and evaluated the proportion of these that surveyors detected; detection tests were done for all surveyors that conducted multiple collision surveys. We incorporated data from these trials into a statistical estimator^70^, implemented in the R package carcass^71^, to quantify daily carcass persistence probability, surveyor detection probability, and ultimately, adjusted estimates of carcass counts that account for these biases. Because only 2 of 14 (14%) month-residency class combinations had values of zero, we did not use zero-inflated regression for this analysis of monthly/seasonal patterns of collisions; however, because data were overdispersed we employed a negative binomial distribution (function ‘glm.nb’ in R package ‘MASS’) with an offset for number of surveys to account for varying effort. We chose a negative binomial distribution instead of a quasi-Poisson distribution because we sought for all of our models to be relatively similar structurally (the above-described Poisson models are a special case of negative binomial models). For the analysis of bias-adjusted mortality estimates, we rounded values to the nearest integer because the negative binomial is a discrete probability distribution. Potential predictors included Month (coded numerically as 4 = April, 5 = May, etc.), Season (categorical: spring, summer, or fall), ResStatus (i.e., residency status of individual birds coded categorically: migrant or resident), and interactions between Season and ResStatus and between Month and ResStatus. As Month and Season were conceptually and statistically correlated, we did not include both together in any models. For both diel and monthly analyses, the levels of categorical variables against which we made comparisons were fall (Season), April (Month), and migrant (ResStatus).

## Supplementary Information

Supplementary Information.

## Data Availability

The datasets generated and/or analyzed during the current study are available from the corresponding author on reasonable request.
