# Supplementary material for: Multi-scale temporal variation in bird-window collisions in the central United States
Source: Sci Rep. 2021 May 26;11:11062. doi: 10.1038/s41598-021-89875-0 (PMC8155105; doi:10.1038/s41598-021-89875-0)
Supplement: Supplementary file 1 — Supplementary Information. [file 41598_2021_89875_MOESM1_ESM.docx]

Supplemental material for

**Multi-scale temporal variation in bird-window collisions**

**in the central United States**

by Corey S. Riding, Timothy J. O’Connell, and Scott R. Loss

The supplemental material includes four tables and one figure.

**Supplementary Table S1**. Full selection results for zero-inflated Poisson regression models that included factors potentially affecting numbers of fatal bird collisions found in morning, midday, and evening collision surveys in Stillwater, Oklahoma, USA in 2015-2016. As described in the text, models were excluded from consideration if they were more complex versions of higher ranking models (i.e., models that contained uninformative parameters).

| **Logit model** | **Count model** | **K^a^** | **ΔAICc^b^** | **Weight^c^** | **Excluded?** |
| --- | --- | --- | --- | --- | --- |
| SurveyTime | Season | 7 | 0.0 | 0.195 | No |
| SurveyTime | Null | 5 | 1.0 | 0.119 | No |
| SurveyTime*Season | Null | 9 | 1.3 | 0.104 | Yes |
| SurveyTime+Season | Null | 7 | 1.4 | 0.094 | Yes |
| Null | SurveyTime+Season | 7 | 1.6 | 0.087 | No |
| SurveyTime | SurveyTime+Season | 8 | 2.0 | 0.073 | Yes |
| Season | SurveyTime | 7 | 2.4 | 0.059 | No |
| Null | SurveyTime | 5 | 2.5 | 0.056 | No |
| SurveyTime | SurveyTime | 6 | 3.0 | 0.044 | Yes |
| SurveyTime*Season | SurveyTime | 10 | 3.2 | 0.039 | Yes |
| SurveyTime+Season | Season | 9 | 3.4 | 0.036 | Yes |
| SurveyTime+Season | SurveyTime | 8 | 3.5 | 0.034 | Yes |
| SurveyTime*Season | Season | 11 | 4.3 | 0.023 | Yes |
| SurveyTime+Season | SurveyTime+Season | 10 | 5.4 | 0.013 | Yes |
| Null | SurveyTime*Season | 9 | 5.4 | 0.013 | Yes |
| SurveyTime | SurveyTime*Season | 10 | 5.9 | 0.010 | Yes |
| Null | Season | 6 | 17.1 | <0.001 | No |
| Season | Null | 6 | 17.9 | <0.001 | No |
| Null | Null | 4 | 18.1 | <0.001 | No |
| Season | Season | 8 | 19.6 | <0.001 | Yes |

^a^ Number of model parameters

^b^ Difference in AICc value between model and top model

^c^ AICc Weight - relative support for model

**Supplementary Table S2**. Full selection results for zero-inflated Poisson regression models that included factors potentially affecting numbers of non-fatal bird collisions found in morning, midday, and evening collision surveys in Stillwater, Oklahoma, USA in 2015-16. As described in the text, models were excluded from consideration if they were more complex versions of higher ranking models (i.e., models that contained uninformative parameters).

| **Logit model** | **Count model** | **K^a^** | **ΔAICc^b^** | **Weight^c^** | **Excluded?** |
| --- | --- | --- | --- | --- | --- |
| Null | SurveyTime | 5 | 0.0 | 0.435 | No |
| SurveyTime | Null | 5 | 1.6 | 0.191 | No |
| SurveyTime | SurveyTime | 6 | 2.0 | 0.159 | Yes |
| Season | SurveyTime | 7 | 2.9 | 0.102 | Yes |
| Null | SurveyTime+Season | 7 | 3.4 | 0.080 | Yes |
| SurveyTime | SurveyTime+Season | 8 | 5.4 | 0.029 | Yes |
| SurveyTime | SurveyTime*Season | 10 | 9.4 | 0.004 | Yes |
| Null | Null | 4 | 20.1 | <0.001 | No |
| Season | Null | 6 | 23.1 | <0.001 | Yes |
| Null | Season | 6 | 23.5 | <0.001 | Yes |

^a^ Number of model parameters

^b^ Difference in AICc value between model and top model

^c^ AICc Weight - relative support for model

**Supplementary Table S3**. Full selection results for negative binomial regression models that included factors potentially affecting (a) monthly counts of total fatal collisions, (b) monthly counts of carcasses excluding feather piles, and (c) bias-adjusted monthly counts of fatal collisions, from collision surveys in Stillwater, Oklahoma, USA. As described in the text, models were excluded from consideration if they were more complex versions of higher ranking models (i.e., models that contained uninformative parameters).

(a)

| **Variables** | **K^a^** | **ΔAICc^b^** | **Weight^c^** | **Excluded?** |
| --- | --- | --- | --- | --- |
| ResStatus * Season | 7 | 0.0 | >0.999 | No |
| Null | 2 | 105.8 | <0.001 | No |
| Season | 4 | 108.2 | <0.001 | Yes |
| Month | 3 | 108.5 | <0.001 | Yes |
| ResStatus + Season | 5 | 108.9 | <0.001 | Yes |
| ResStatus | 3 | 108.9 | <0.001 | Yes |
| ResStatus * Month | 5 | 110.2 | <0.001 | Yes |
| ResStatus + Month | 4 | 112.5 | <0.001 | Yes |

(b)

| **Variables** | **K^a^** | **ΔAICc^b^** | **Weight^c^** | **Excluded?** |
| --- | --- | --- | --- | --- |
| ResStatus * Season | 7 | 0.0 | >0.999 | No |
| Season | 4 | 94.5 | <0.001 | No |
| Null | 2 | 97.1 | <0.001 | No |
| ResStatus + Season | 5 | 98.2 | <0.001 | Yes |
| ResStatus * Month | 5 | 99.0 | <0.001 | Yes |
| ResStatus | 3 | 99.8 | <0.001 | Yes |
| Month | 3 | 100.0 | <0.001 | Yes |
| ResStatus + Month | 4 | 103.1 | <0.001 | Yes |

(c)

| **Variables** | **K^a^** | **ΔAICc^b^** | **Weight^c^** | **Excluded?** |
| --- | --- | --- | --- | --- |
| ResStatus * Season | 7 | 0.0 | >0.999 | No |
| ResStatus + Season | 5 | 16318 | <0.001 | No |
| Season | 4 | 16504 | <0.001 | No |
| ResStatus * Month | 5 | 16876 | <0.001 | No |
| ResStatus + Month | 4 | 18031 | <0.001 | No |
| ResStatus | 3 | 18048 | <0.001 | No |
| Null | 2 | 18230 | <0.001 | No |
| Month | 3 | 18232 | <0.001 | Yes |

^a^ Number of model parameters

^b^ Difference in AICc value between model and top model

^c^ AICc Weight - relative support for model

**Supplementary Table S4**. Negative binomial model coefficients and standard errors for factors potentially affecting (a) monthly counts of total fatal collisions, (b) monthly counts of carcasses excluding feather piles, and (c) bias-adjusted monthly counts of carcasses, from collision surveys in Stillwater, Oklahoma, USA.

(a)

| **Factor** | **Coefficient** | **Standard Error** |
| --- | --- | --- |
| Intercept | 3.58 | 0.13 |
| Status-Resident | -1.44 | 0.20 |
| Season-Spring | -0.13 | 0.19 |
| Season-Summer | -3.30 | 0.25 |
| Status-Resident * Season-Spring | 1.17 | 0.28 |
| Status-Resident * Season-Summer | 4.56 | 0.32 |

(b)

| **Factor** | **Coefficient** | **Standard Error** |
| --- | --- | --- |
| Intercept | 3.57 | 0.14 |
| Status-Resident | -1.78 | 0.22 |
| Season-Spring | -0.14 | 0.20 |
| Season-Summer | -3.29 | 0.26 |
| Status-Resident * Season-Spring | 1.31 | 0.30 |
| Status-Resident * Season-Summer | 4.55 | 0.33 |

(c)

| **Factor** | **Coefficient** | **Standard Error** |
| --- | --- | --- |
| Intercept | 3.85 | 0.02 |
| Status-Resident | -1.77 | 0.02 |
| Season-Spring | -0.30 | 0.02 |
| Season-Summer | -3.57 | 0.02 |
| Status-Resident * Season-Spring | 1.31 | 0.02 |
| Status-Resident * Season-Summer | 4.63 | 0.03 |


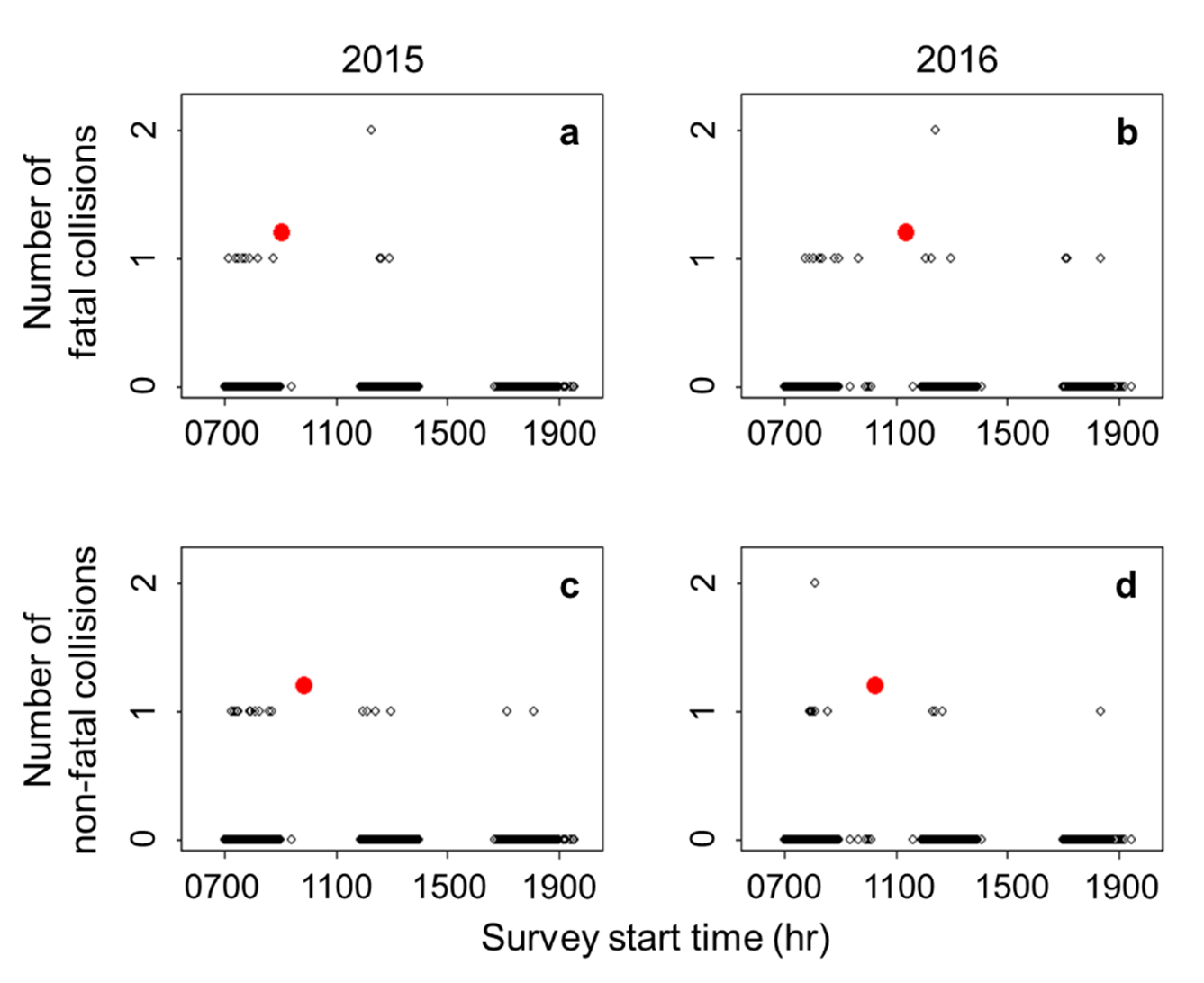


**Supplementary Figure S1**. Variation in bird-window collisions relative to time of day. Number of fatal bird collisions including feather piles (a, b) and number of non-fatal bird collisions (i.e., stunned birds or birds observed to collide and then fly away) (c, d) found on collision surveys with different start times for 2015 and 2016 in Stillwater, Oklahoma, USA. Red dots indicate annual mean start time of surveys with at least one collision counted.
